# A Review of Research Progress on Ti(C,N)-Based Cermet Binder by Intermetallic Compounds and High-Entropy Alloys

**DOI:** 10.3390/ma17030675

**Published:** 2024-01-30

**Authors:** Liang Wang, Jingfei Bai, Yanghe Wang, Zhengxing Men

**Affiliations:** Chengdu Aeronautic Polytechnic, Chengdu 610100, China

**Keywords:** Ti(C,N), ceramic, binder, intermetallic compounds, high-entropy alloys

## Abstract

Ti(C,N)-based cermet is a kind of composite material composed of a metal binder phase and a Ti(C,N)-hard phase, which is widely used in the fields of cutting machining and wear-resistant parts due to its high hardness, good toughness, wear resistance, and chemical stability. In recent years, the research on the replacement of traditional Ni, Co, and Fe binder phases by novel binder phases such as intermetallic compounds and high-entropy alloys has made remarkable progress, which significantly improves the mechanical properties, wear resistance, corrosion resistance, and high-temperature oxidation resistance of Ti(C,N)-based cermets. This paper reviews the latest research results, summarizes the mechanism of the new binder to improve the performance of metal–ceramics, and looks forward to the future research directions.

## 1. Introduction

**Figure 1:**
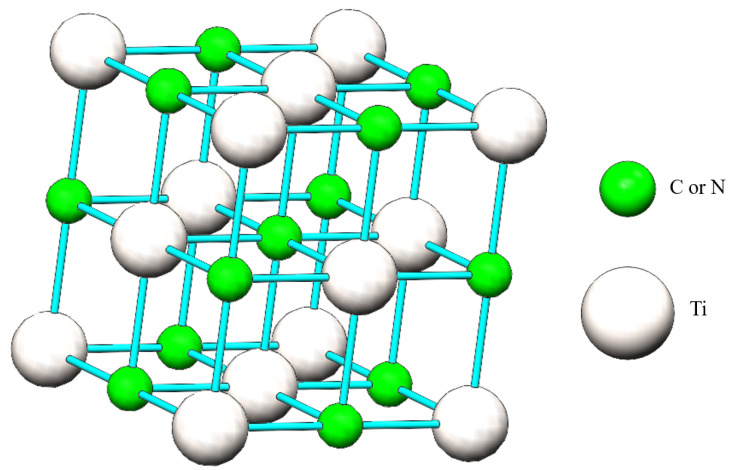
The lattice structure of the TiC or TiN crystal.

Ti(C,N)-based cermet is a kind of metal–ceramic material prepared by the powder metallurgy method using TiC, TiN, and Ti(C,N) as the matrix composition [1,2,3,4], the titanium carbide structure, as shown in Figure 1, Fe-Co-Ni as the binder [5,6,7,8], and other transition metal carbides [9,10,11,12]. Because of its high hardness, excellent wear resistance, low coefficient of friction, and good chemical stability, Ti(C,N)-based cermet has become an important alternative material to cemented carbide and is widely used in cutting and grinding tools [13,14,15,16,17]. However, the traditional Ni, Co, and Fe binder phases are prone to softening at high temperatures [18], leading to rapid tool wear and oxidation [19] and reducing the service life of cermets. Therefore, the development of a new binder to improve the performance of Ti(C,N)-based cermets has become a hot spot in current research [20,21,22].

**Figure 2:**
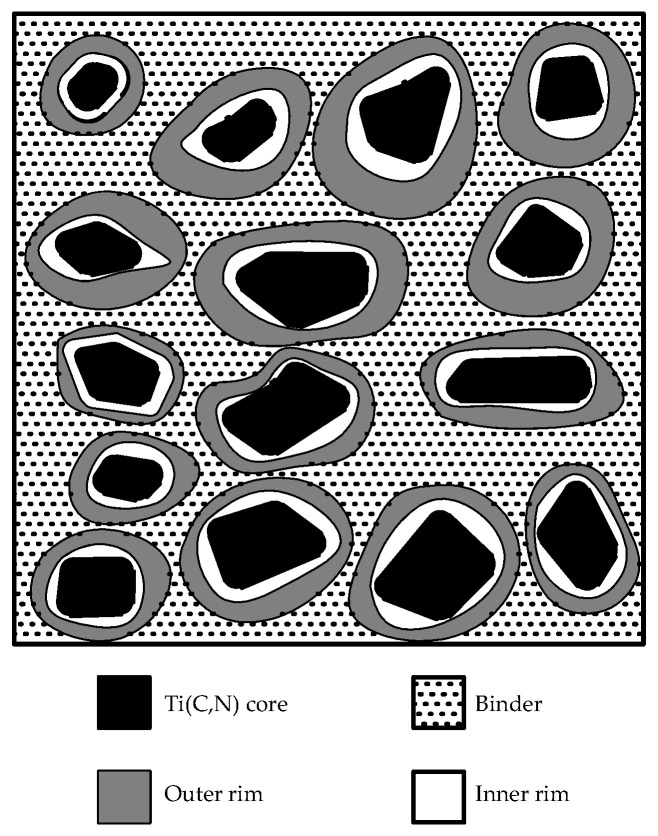
Schematic structure of the Ti(C,N)-based cermet.

The conventional binder phases Fe, Co, and Ni have good wettability to Ti(C,N), and Ti(C,N) has a certain solubility in them, which can ensure the high densification of the sintered cermet [23,24,25,26,27,28,29,30,31]. The microstructure of Ti(C,N)-based cermet is shown in Figure 2. The organizational structure consists of a binder, a hard phase, and an annular phase, and the hard phase and annular phase form a typical “core–ring” structure, while the metal binder is distributed around the “core–ring” structure. The black core phase is the hard phase [32], which consists of the original undissolved TiC or Ti(C,N) particles and mainly affects the hardness of Ti(C,N)-based cermet materials; the ring phase is divided into a white inner ring and a gray outer ring, whose chemical composition is similar to that of the black core phase, but with different elemental concentrations; the inner ring phase, formed by the “dissolution–precipitation” mechanism during the solid-phase sintering stage, is rich in metal binders. The inner ring phase is formed in the solid-phase sintering stage by the “dissolution–precipitation” mechanism and is rich in Ti, while the outer ring is formed in the liquid-phase sintering stage and has less Ti. The ring phase is a transition phase, which can effectively improve the wettability of the binder to the hard phase, inhibit the merging of hard phase particles and, then, grain refinement, so that the Ti(C,N)-based cermet microstructure is uniform. By observing the composition and microstructure of the ceramic and binder phases before and after sintering, the previous authors found that the microhardness increases with the increase in the continuity of the Ti(C,N) hard phase and the decrease in the particle size. The particle size of the hard phase is smaller because the binder phase hinders the ceramic growth during liquid-phase sintering, i.e., it indicates that the binder phase has a very strong influence on the hardness of the cermet [33,34,35,36]. The traditional binder is mainly composed of the integration of Ni, Co, and other metal elements, and the distribution of the binder is conducive to the enhancement of the toughness and strength of Ti(C,N)-based cermet. However, since the category and content of the binder largely determine the comprehensive performance of metal–ceramics [37,38,39,40,41], in order to improve the performance of metal–ceramics, many scholars have solved the problem by modulating the composition of the metal binder. With the improvement of the difficulty of workpiece processing technology and the harsh environment of material use, the shortcomings of Co’s and Ni’s poor high-temperature performance gradually appeared, and there is an urgent need to develop a new binder cermet [42,43].

**Figure 3:**
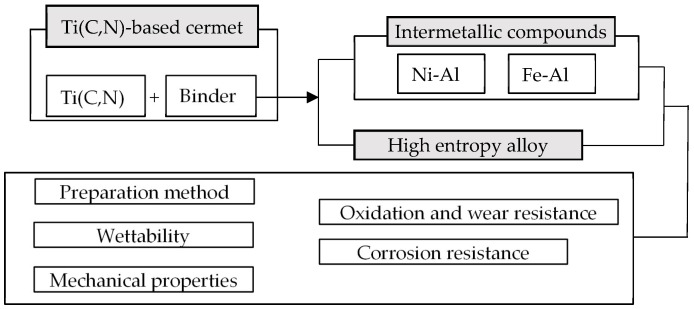
Review frame diagram.

At present, There has been a great deal of research on new binders for cermets, which mainly include intermetallic compounds and high-entropy alloys. Intermetallic compounds are compounds composed of two or more metal elements, with excellent mechanical properties and high temperature stability. High-entropy alloys are alloys composed of five or more metal elements with excellent properties such as high strength, high hardness, wear resistance, and oxidation resistance. In this review, we will start from the Ti(C,N)-based cermets with these two binders and elaborate on the relevant research progress on their microstructure, mechanical properties, wear resistance, high-temperature oxidation resistance, and corrosion resistance to explore the mechanism of the influence of the new binders on the cermets’ organization and properties [44,45,46]. The development of new binder phases for Ti(C,N)-based cermets is also prospected, with a view toward providing a theoretical basis for the preparation of new cermets with excellent comprehensive performance. The structural framework of this paper is shown in Figure 3, which discusses the preparation method, wettability, mechanical properties, oxidation resistance, and corrosion resistance of the binder between intermetallic compounds and high-entropy alloys.

## 2. Ti(C,N)-Based Cermets with Intermetallic Compounds as the Binder

Ti(C,N)-based cermets with Ni-Al and Fe-Al intermetallics (Ni_3_Al, NiAl, FeAl, etc.) as the binder phase have received wide attention recently [47,48]. These intermetallics are able to maintain strength and stiffness at high temperatures. Among them, Ni_3_Al is the most-widely studied. Ni_3_Al is mainly an L12-type face-centered cubic structure at room temperature [49,50], and its strength increases with increasing temperature in a certain temperature range. Ni_3_Al will form an Al_2_O_3_ passivation layer at high temperature [51], which makes the metal–ceramics have good resistance to high-temperature oxidation [52].

### 2.1. Ni-Al Intermetallic Compounds as Binders

Transition metal aluminides Ni-Al exhibit good antioxidant properties because of their ability to form a dense protective layer of alumina in high-temperature environments. In addition, they have attracted much attention due to their low density, dystectic point, good thermal conductivity, and high-temperature strength [53].

#### 2.1.1. Preparation Method of Ni-Al Intermetallic Compounds as Binders

Intermetallic compounds are prepared using the method of pre-alloyed intermetallic powders [54,55], and the other is reflected by in situ mechanical alloying. Specific processing techniques include vacuum liquid-phase sintering (LPS) [56,57], high-frequency induction sintering (HFIS) [54], spark plasma sintering (SPS) [58], and hot pressing (HP) [59].

**Figure 4:**
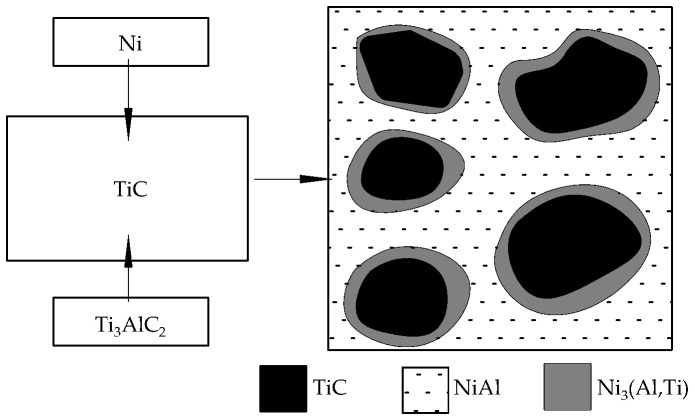
NiAl binder molding process.

A novel TiC/Ni_3_(Al,Ti)-NiAl composite combining excellent flexural strength, high fracture toughness, and high Vickers hardness was prepared by reactive hot pressing and sintering of the powders of Ti_3_AlC_2_ and Ni at 1500 °C and 30 MPa. At high temperatures, the molten nickel promoted the decomposition of Ti_3_AlC_2_, and the released binder titanium atoms reacted with the nickel to form Ni_3_(Al,Ti) and NiAl alloys, which was accompanied by a lattice structure transformation from a hexagonal lattice Ti_3_AlC_2_ to a cubic lattice TiC. The NiAl binder molding process is shown in Figure 4. At room temperature, the hardness, flexural strength, and fracture toughness of the composites were 9.9 ± 0.35 GPa, 665 ± 26 MPa, and 10.23 ± 0.4 MPa·m^1/2^. As the temperature was increased from 600 °C to 800 °C, the flexural strength and fracture toughness increased and reached a maximum value of 775 ± 25 MPa and 11.6 ± 0.4 MPa·m^1/2^, respectively, at 800 °C. By analyzing the fracture mechanism, it was found that the TiC was transformed into a cubic lattice Ti_3_(Al,Ti), and interface debonding and cleavage of the binder NiAl compound were found to be the main fracture causes [60].

During the sintering process, the Ni_3_Al phase was formed in the metal–ceramics due to the addition of AlN. When the addition of AlN was 2.5 wt.%, it was found that the incorporation of AlN hindered the dissolution recrystallization process, leading to a significant increase in grain size at the core edge. In addition, because of the decomposition of AlN and the release of N_2_, the porosity of the metal–ceramics increased significantly, resulting in a gradual decrease in hardness and transverse fracture strength when the addition of AlN exceeded 2.5 wt.% [61].

#### 2.1.2. Wettability of Ni-Al Intermetallic Compound Binder

The microstructure of Ti(C,N)-based cermets with intermetallic compounds as the binder is similar to that of conventional cermets, which mainly consists of a hard phase and a binder. However, the wettability of intermetallic compounds on Ti(C,N) is not as good as that of Ni, and a higher content is required for better wetting of Ti(C,N). For example, Ti(C,N)-Ni_3_Al cermets with a Ni_3_Al content of 20–40 vol.% were prepared by the fusion infiltration method, and the hard-phase particles hardly grew [62]. Unpenetrated regions are prone to appear when the Ni_3_Al content is low. At a concentration of 40 vol.%, a denser organization is obtained.

Ti(C,N)-40 vol.% Ni_3_Al cermets were prepared by vacuum sintering, and although the densities were higher than those of the fusion infiltration method, there was grain growth. The hard-phase morphology of Ti(C,N)-based cermets with Ni_3_Al as the binder is more rounded than that of conventional cermets. This is due to the lower diffusion coefficient of each element in Ni_3_Al than in Ni, which slows down the dissolution precipitation and grain growth of the hard phase of Ti(C,N) during the liquid-phase sintering process [63].

In Ti(C,N)-Ni_3_Al cermet, the addition of Mo_2_C, WC, and other carbides can improve the wettability of the hard phase of the binder, and the organization will form a “core–ring” structure [64]. The “core–rim” structure of Ni_3_Al binder cermets has a more-rounded core phase than that of Ni binder cermets, and the ring phase around the black core is very thin or even absent. The ring phase is formed by the dissolution and precipitation of Ti(C,N) and other carbides during liquid-phase sintering, which is controlled by diffusion. Studies have shown that the solubility of Ti, W, Mo, and other elements in Ni_3_Al is much lower than that of Ni, and the diffusion coefficients of each element in Ni_3_Al are low. Therefore, Ni_3_Al as the binder phase can inhibit the abnormal growth of the Ti(C,N)-based cermet core phase to make it rounded, and it can make the thickness of the annular phase thinner to reduce the effect of the low hardness of the annulus on the hardness of the cermet [65,66].

Mo_2_C improved the wettability and densification of the cermets. TiC_0.3_N_0.7_-Ni_3_Al-Mo_2_C cermets were prepared by vacuum sintering. Additionally, the addition of Mo_2_C efficiently decreased the grain size of Ti (C_0.3_, N_0.7_), which was achieved by decreasing the solubility of the carbon nitride phase in the Ni_3_Al binder [67].

Ni_3_Al binder cermet exhibits the rim–core texture with carbide grains bonded to the edges embedded in the Ni_3_Al binder. As the WC increases, the TiC grains refine, and the rim gradually becomes thicker and complete. The interface between the core and the edges shows a perfectly consistent feature. The W-rich edge constitutes a perfect coherence between the hard phase and the Ni_3_Al binder phase. With the increase of WC content, the densification of the metal–ceramics increases, and the hardness firstly increases and, then, decreases [68].

#### 2.1.3. Mechanical Properties of Ni-Al Intermetallic Compounds as Binders

With the increase of the component Ni_3_Al, the hardness of Ti(C,N)-based cermet decreases, but the toughness increases significantly. The best overall mechanical properties are obtained when the Ni_3_Al content is 30 mass.% [69]. At a lower content of Ni_3_Al, it cannot circulate sufficiently after melting, and holes will be formed. The fracture surface consists of tearing prongs and tearing surfaces produced by plastic deformation of the binder, as well as pits and disintegration surfaces produced by dislodging and disintegration of the hard phase [70,71]. Conversely, the Ni_3_Al content is increased to 30 mass.%, the fracture hole almost disappears, and the fracture surface is mainly composed of tearing ribs and tearing surfaces generated by the augmentation of the binder, as well as tough pits formed by the detachment of the hard phase [72,73]. This indicates that this apparent plastic deformation of the binder can consume the energy of the externally acting force, thus improving the toughness of the material.

With the increase of Ni_3_Al content and the increase of the sintering temperature, the density of the metal–ceramics gradually increases, while the porosity decreases accordingly. In addition, the transverse rupture strength (TRS) and fracture toughness also show a tendency to increase with the Ni_3_Al content. The TRS increases with increasing sintering temperature. However, the TRS reaches a peak when the sintering temperature reaches 1450 °C, after which it decreases with a further increase in temperature. Similarly, the HRA of the metal–ceramics increases and, then, decreases with increasing sintering temperature and also peaks at 1450 °C [74,75].

#### 2.1.4. Oxidation and Wear Resistance of Ni-Al Intermetallic Compounds as Binders

The high hardness of the intermetallic compound Ni_3_Al itself gives Ti(C,N)-Ni_3_Al cermets higher wear resistance compared to conventional cermets [76,77]. The wear behavior of Ti(C,N)-Ni_3_Al cermets was investigated by preparing them by fusion infiltration and vacuum sintering methods, and the samples were subjected to reciprocal wear tests using a friction wear tester. These behaviors include surface binder wear, plastic deformation of the subsurface binder, strain accumulation in the ceramic particles, and fracture, fragmentation, and detachment of the ceramic particles. The wear of Ti(C,N)-based Ni_3_Al cermet changes from two-body abrasive wear to three-body abrasive wear, with adhesive wear gradually appearing. On the other hand, oxidative wear begins to appear, and the high hardness Al_2_O_3_ is formed on the surface of the cermet to improve its wear resistance. Qi [61] prepared Ti(C,N)-Ni cermets with the addition of AlN and found that the addition of AlN generates Ni_3_Al or Ni_3_(Al,Ti) diffusely reinforced phases, which improves the wear-resistant properties of the materials.

**Figure 5:**
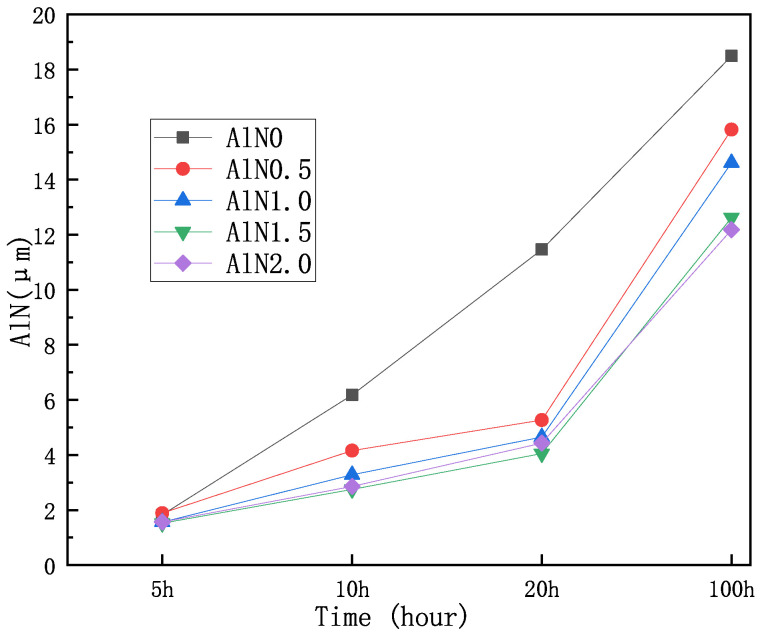
Nitriding layer thickness of all samples with various oxidation times [79].

During oxidation under high-temperature conditions, the Ni_3_Al binder is oxidized to form the double-oxide NiAl_2_O_4_, which can effectively inhibit the diffusion of O_2_ in the oxide and, thus, reinforce the oxidation resistance of the metal–ceramic binder [78]. Ti in the hard phase is primarily oxidized to TiO_2_, while W and Mo are oxidized to WO_3_ and MoO_3_, respectively. The cross-section morphology of oxidized Ni_3_Al binder cermet consists of an oxide layer (OL), a transformation layer (TL), and a matrix. Some pores can be observed in the OL, and slight pores exist in the TL. By observing the SEM images of five Ti(C,N)-20 wt.%, WC-10 wt.%, Mo_2_C-2 wt %, TaC-7.5 wt.%, Ni-7.5 wt.%, and Co-xwt.%AlN with an AlN content x of 0, 0.5, 1.0, 1.5, and 2.0, respectively, with different oxidation times, it can be seen that, for each metal–ceramic, the overall thickness variation of the oxide layer on the surface is inversely proportional to the amount of Al added. The thicknesses are shown in Figure 5. This indicates that the addition of Al greatly improves the oxidation resistance of the metal cermets [79]. The thickness of the OL of Ni_3_Al binder cermet increases with increasing oxidation temperature and holding time. During the oxidation process, elements such as Al and Ti will diffuse outward from the inside of the cermet to the OL, while O_2_ will diffuse inward to the inside of the cermet to form an oxygen-permeable zone, i.e., the transition layer [80].

In the static oxidation experiments at 900 °C, the metal cermets without AlN exhibited the largest mass gain. When 2.5 wt.% of AlN was added, the mass gain value of the metal cermets decreased significantly, and the oxidation kinetic curves maintained a similar quasi-parabolic law as those of the AlN-free metal cermets. However, further addition of up to 7.5 wt.% AlN led to a decrease in the oxidation resistance, which is mainly due to the presence of macropores. In this case, the oxidation kinetic curve becomes a linear law. During the oxidation process, the hard-phase (TiC,TiN) is mainly oxidized to TiO_2_, while the binder Ni is transformed to its oxides Ni_2_O_3_, Ni_0.75_Ti_0.125_O, Ni_3_TiO_7_, and NiTiO_3_, as well as NiMoO_4_, NiAl_2_O_4_, and Ni_3_TiO_7_ in the metal–ceramics. It is worth noting that, when adding 2.5 wt.% of AlN, the intermediate oxidation products of the Ti(C,N) hard phase, i.e., TiO_0.19_, C_0.53_, N_0.32_, and Ni_3_Al, remained stable after 4 h of residence. This result showed that the addition of 2.5 wt.% AlN inhibited the diffusion of oxygen into the metal–ceramics, thus improving the oxidation resistance of the metal–ceramics [61].

#### 2.1.5. Corrosion Resistance of Ni-Al Intermetallic Compounds as Binder Phases

The binder Ni often requires the introduction of Cr to increase its corrosion resistance in Ti(C,N)-based cermets. The use of Ni_3_Al instead of Ni can also improve the corrosion resistance of cermets. The study of the effect on the corrosion behavior of Ti(C,N)-based cermet in 3.5 mass.% NaCl solution showed that, when the content of Ni_3_Al was less, the corrosion surface was prone to cratering and the corrosion resistance was poor. The corrosion of metal–ceramics is mainly due to the dissolution of Ni_3_Al during oxidative attack in NaCl solution. Metal–ceramics with low binder content have poor densification, many pores, and an uneven Ni_3_Al concentration in the surface area exposed to the corrosion medium; thus, pits are easily formed after corrosion. TiC-Ti(C_0.5_,N_0.5_)-WC-Mo-C-Ni_3_Al cermets were made by the vacuum sintering method and tested for their corrosion resistance in NaOH solution. It was found that the corrosion of the cermets was primarily caused by the dissolution of the ceramic phase. With the increase of Ni_3_Al content, the corrosion potential increased and the current density decreased, showing that the corrosion resistance is better with the increase of the Ni_3_Al binder.

Based on the electrochemical characteristics of Ni_3_Al and TiC- and TiCN-based cermets, the dissolution rate of the binder during the corrosion process is significantly less dependent on the Ni_3_Al content than on the WC-Co hard alloys. Unlike Co, the dissolution of the Ni_3_Al adhesive in corrosive media has no effect on the pH value of the solution. Therefore, increasing the content of the Ni_3_Al intermetallic binder can be considered to improve the ductility of cermets without affecting the oxidation characteristics and performance of the entire composite material [81].

The corrosion rates of TiC-Ni_3_Al and Ti(C,N)-Ni_3_Al cermets are lower than those of commercially available WC-Co equivalents and are dependent on the nitrogen content of the Ti(C,N) alloy’s composition [82].

With the increase of the Ni_3_Al content, the corrosion potential showed an increasing trend, while the current density decreased, which indicated that the increase of the Ni_3_Al bound phase could effectively enhance the corrosion resistance of the material. However, when Ti(C_0.5_,N_0.5_) is added, the corrosion potential decreases and the current density increases, which may indicate that the addition of Ti(C_0.5_,N_0.5_) reduces the corrosion resistance of the material to some extent. Corrosion experiments have been performed using sulfuric acid and nitric acid. It was found that, in H_2_SO_4_ solution corrosion, the corrosion potential and current density decreased with the increase of the Ni_3_Al content. In NaOH solution corrosion, with the increase of the Ni_3_Al content, the corrosion potential increases and the current density decreases [83].

### 2.2. Fe-Al Intermetallic Compounds as Binders

Due to the relatively low cost of Fe-Al compounds, it has become a preferred material for researchers [84,85]. Among these, Fe_3_Al (with an aluminum content of about 23–36 at.%) and FeAl (with an aluminum content of about 36–48 at.%) are the two substances that have received the most attention [86,87,88,89,90].

#### 2.2.1. Preparation Method of Fe-Al Intermetallic Compounds as Binders

Powdered Fe, Al, Ti, and C were used as raw materials. Dosing was carried out according to the weight ratio of Fe to Al of 84.15:15.85, and an alloy of Fe-28 at.% Al was produced by the reaction of Fe with Al. During the mechanical alloying process, a ball mill was used to mix and grind the elemental Ti, C, Fe, and Al powders. A ball mill jar and a stainless steel ball with a diameter of 9.525 mm were used as the grinding tools. The mixed powders were milled separately in an argon atmosphere at a rate of 320 rpm for 48 h. Subsequently, the mechanically milled powders were poured into graphite molds and pressed in a hot pressing process. During hot pressing, the samples were heated to 1250 °C in vacuum at a rate of 10 °C/min and, then, pressed at 50 MPa for 30 min. Finally, the samples were cooled to room temperature in vacuum [91]. The mechanical alloying process of the Fe, Al, Ti and C powders used involved repeated cold welding and fracturing of the powder particles [92]. After 6 h of milling, some of the Ti began to react with the C on the surface of the agglomerated milled powder. As the grinding process progressed, the reaction between the two elements gradually extended to the interior of the powder, while reactions between Fe and Al also began to occur [93]. After 48 h of milling, it was determined that the powder obtained from milling contained only two components, TiC and Fe_3_Al. the reaction between C and Ti was considered to be more preferred than that between Fe and Al because of its lower reaction energy [94,95].

Another method is to add Fe powder to the high-purity TiC material, pressing Fe-doped TiC powders, then Al melt penetration. This preparation method effectively reduces the reactivity of Al and TiC during Al melt penetration, which helps to press the TiC preforms, thus successfully preparing a lightweight TiC-(Fe-Al) composite. Also, the method successfully avoided the generation of other unwanted phases. The composite was characterized by low density, low porosity, and high hardness, and the presence of the Al_4_C_3_ phase was not detected by X-ray diffraction. This indicates that the material maintained a good shape during the infiltration process without any significant phase change or deformation. This TiC-(Fe-Al) composite material has a good application prospect, especially in the application areas where high hardness, low density, low porosity, and high shape stability are required [96].

#### 2.2.2. Wettability of Fe-Al Intermetallic Compound Binder

Submicron Fe_3_Al(Ti) intermetallic compounds reinforced by TiC nanoparticles can be successfully prepared by mechanical alloying (MA) and hot press sintering. In this process, TiC particles with sizes in the range of 50 to 200 nm can be uniformly dispersed in the Fe_3_Al matrix. Larger TiC particles at the submicron level are mainly located at grain boundaries, while smaller particles are located within the matrix grains [97].

#### 2.2.3. Mechanical Properties of Fe-Al Intermetallic Compounds as Binders

The Fe_3_Al(10Ti)/40 vol.% TiC composites exhibited high bending strength and hardness, respectively. This excellent performance is mainly attributed to the reinforcing effect of the TiC nanoparticles, which is the main reinforcing mechanism of the composites [97]. In addition, the incorporation of Ti facilitates the improvement of the hardness and room temperature bending strength of the composites through the solid solution hardening effect and ordered strengthening effect [98]. Fe_3_Al(Ti)/TiC-based cermets were prepared and analyzed. The hardness increased significantly with the increase of the TiC content, while the addition of Ti was favorable to improve the hardness of Fe_3_Al/TiC composites. For example, the hardness increased from 87 HRA for Fe_3_Al(0Ti)/40 vol.% TiC composites to 90 HRA for Fe_3_Al(10Ti)/40 vol.% TiC composites. The flexural strength of the metal–ceramics increased with the increase of the TiC content [99], which was mainly affected by three factors: the reinforcement effect brought by the addition of TiC, the densification effect of the composites, and the grain size effect. The presence of TiC particles at the grain boundaries inhibits the grain growth of Fe_3_Al, which results in the refinement of the grain structure of the composites and provides an additional contribution to the flexural strength. The TiC particles may act as barriers to dislocation motion during deformation, which results in a strengthening effect on the Fe_3_Al matrix. However, the fracture toughness of the composites decreases with increasing TiC content due to the inhibition of ductility by brittle TiC [97,100].

In terms of the mechanical properties of Fe_3_Al, the introduction of the alloying elements V, Cr, and Ni led to an increase in the alloy Pugh’s ratio, showing an increase in ductility. However, the addition of Mn, Co, and Cu decreased the ductility of the alloy. In addition, the smallest elastic anisotropy is induced by V compared to the other alloying elements. V contributes to the stabilization of Fe_3_Al-V alloys by lowering the magnetic moment of the system and reducing the spin asymmetry of the iron atoms around V. The addition of Cr and Ni leads to an increase in the elastic anisotropy of the alloys. In contrast, Ni is observed to have a partially filled antibonding state, which adversely affects the structural stability of its Fe_3_Al-Ni [101].

#### 2.2.4. Oxidation and Wear Resistance of Fe-Al Intermetallic Compounds as Binders

The OL of the WC powder can be effectively reduced by heat treating the powder, and the reduction of the oxygen content can significantly improve the thermal conductivity of the WC-FeAl composites and effectively inhibit the role of Al_2_O_3_ generation [102]. However, there is a complex negative correlation between key mechanical properties such as TRS, hardness, and thermal conductivity. This suggests that the pursuit of good thermal performance cannot be achieved by reducing the material hardness and transverse fracture strength. Therefore, we need to find a suitable balance in the selection and processing of WC powders to achieve both good thermal conductivity and maintain excellent mechanical properties [103].

#### 2.2.5. Corrosion Resistance of Fe-Al Intermetallic Compounds as Binder Phases

The relative order of the corrosion resistance of cermets with different hard particles (WC, TiB_2_, TiC) with FeAl as the binder is as follows: FeAl-WC > FeAl-TiB_2_ > FeAl-TiC. The corrosion resistance is closely related to the microstructure and material removal mechanism of the cermets. FeAl-80v/oTiC cermets consist of large (<10 μm) TiC particles, which tend to fracture during corrosion, leading to relatively high corrosion rates. Due to the low volume fraction and distribution of TiB_2_ particles, the FeAl binder preferentially undergoes wear during the corrosion of FeAl-v/oTiB_2_ cermets. In contrast, the material removal mechanism of FeAl-80v/oWC cermets during corrosion is similar to that of cemented carbide [104].

## 3. Ti(C,N)-Based Cermets with High-Entropy Alloys as the Binder

A high-entropy alloy (HEA) contains more than five elements, and the content of each main element is between 5% and 35% (atomic fraction) with a solid-solution-based or mixed entropy greater than the 1.5R alloy [105,106,107]. Due to the special atomic structure of the multi-principal element components of high-entropy alloys, which are mutual solute and solvent atoms, they exhibit excellent mechanical, oxidation-resistant, and corrosion-resistant properties under the joint action of various microscopic mechanisms. Similar to traditional alloys, high-entropy alloys also have the problem of the strength and plasticity not being easy to match. Therefore, high-entropy alloys with the fcc structure are mainly used as metal–ceramic binders. The properties of high-entropy alloys are superior to those of conventional Ni, Co, and Fe metals, including high-temperature strength, ductility, wear resistance, and oxidation resistance. The phase with Ti(C,N)-based cermet also shows excellent wettability, demonstrating the potential as an excellent binder for Ti(C,N)-based cermet.

### 3.1. Preparation Method of High-Entropy Alloy Binder Phases

The preparation process of the high-entropy alloy binder is consistent with that of the intermetallic compound binder, which both need to go through the steps of batching, ball milling, drying, pressing, and sintering [108]. In the batching stage, various raw materials need to be weighed according to the pre-determined recipe proportions to ensure that the composition and proportions of the alloys meet the desired requirements. Subsequently, the raw materials (comprising the hard phase) are mixed homogeneously through a ball milling process, and the powder particles are refined to improve the flowability of the powder. Next, a drying operation is performed to remove moisture and other volatile components from the powder to prevent porosity and other defects during subsequent sintering [109]. In the pressing stage, the dried powder is poured into a mold and a certain pressure is applied to obtain a blank of the desired shape and density. Finally, in the sintering stage, the billet is heated to a high temperature and held for a period of time to densify and strengthen the alloy [110,111]. Through this series of steps, a high-entropy alloy binder is finally prepared [112].

Using thermodynamic calculations, a mechanism for the formation of the microstructure of TiC-HEA metal–ceramics was proposed. The Vickers microhardness (100 g) of the compacted metal–ceramic material with a binder content of 30 wt.% was in the range of 10–17 GPa and increased with the bbc-to-fcc ratio [113].

Four high-entropy alloys, CoCrCuFeNi, CoCrFeNiV, CoCrFeMnNi, and CoFeMnNiV, were synthesized as alloys by mechanical alloying. The melting points ranged from 1310 °C to 1375 °C. The melting points of the alloys were in the range of 1575 °C. Although high temperatures of 1575 °C are required to obtain the highest densification of metal–ceramics by pressureless sintering, porosity is still present in most metal–ceramics. The highest densification occurs when CoCrFeNiV was used as the binder phase. Because V was added during sintering, elemental V was observed on the core–shell structure of CoCrFeNiV and CoFeMnNiV. Cr and Cu polarization was found in the binder of the high-entropy alloys containing Cr and Cu. The loss of Mn was found in the binder of high-entropy alloys containing Mn, which may be due to its sublimation at the sintering temperature [114].

### 3.2. Wettability of High-Entropy Alloy Binder Phases

The microstructures of the AlCoCrFeNi high-entropy alloy as the binder phase and Ni-Co as the binder phase of Ti(C,N)-based cermet show a “core–ring” structure embedded in the binder phase. However, the outer ring of the high-entropy alloy binder cermet is very thin and almost absent, while the inner ring is thick and incomplete. The outer ring phase is formed by the precipitation of carbides dissolved in the liquid metal. The high mixing entropy of the iso-atomic ratio AlCoCrFeNi binder can significantly reduce the free energy of the system and promote the dissolution of the elements W and Mo in the liquid binder during the liquid-phase sintering process. Due to the slow diffusion effect of the high-entropy alloy, the dissolution precipitation of the elements in the liquid-phase sintering process is slowed down, thus suppressing the grain growth of the hard phase [99].

Two Ti(C,N)-HEA cermets were prepared using MA and gas-atomized (GA) CoCrFeNiCu (HEA) powders as binders. The Ti(C,N)-HEA cermets prepared using CoCrFeNiCu (HEA) powders from MA and GA as binders had a more-uniform core–ring grain distribution and smaller grain size compared to Ni. In addition, the cermet formed finer submicron white coreless grains and fine black core, white inner ring, and gray outer ring grains. It was analyzed that the retarded diffusion effect of the high-entropy alloy significantly suppressed solute diffusion. During the liquid-phase sintering process, the difficult synergistic diffusion of hard elements with different sizes of atoms in the high-entropy alloy binder suppressed the dissolution and precipitation of the hard phases. The large-sized atoms Ta, Mo, and W were not easy to dissolve into the liquid binder and precipitate out, which promoted the independent nucleation of (Ti, Ta, Mo, W)(C, N) particles around the undissolved Ti(C,N) particles and the formation of white coreless grains [115].

The FeCoCrNi-Al-Ti(C,N)-TiB_2_ cermet was characterized by TEM. The “black” phase contained mainly the Co, Cr, Fe, Ni, and Al elements, but its elemental composition changed from an iso-atomic to a near-iso-atomic ratio. The reason was the melting of FeCoCr-NiAl during sintering and the diffusion of some elements. The structure was BCC in SAED mode, which indicates that FeCoCrNiAl does not undergo a phase transition after high-temperature sintering and can exist stably at the grain boundaries of the hard particles [116].

### 3.3. Mechanical Properties of High-Entropy Alloy as Binder

The high-entropy alloy binder cermet exhibits good high-temperature mechanical properties. In the Ti(C,N)-based cermet system, although the composition of the binder phase accounts for a relatively small amount, it plays an important role in fracture toughness and is also the weaker part of the cermet [117]. Therefore, realizing the toughening of the binder phase is an economical and efficient means to enhance the properties of metal–ceramics [118,119]. In numerous studies, high-entropy alloy binder phases have been shown to be effective in improving the properties of the substrate [120]. This is mainly attributed to the high entropy effect, lattice distortion, and retarded diffusion effect of high-entropy alloy binders, which inhibit grain growth and produce solid solution strengthening [121,122].

Cermet of Ti(C_0.7_,N_0.3_) (1–2 μm), WC (2 μm), and Mo_2_C (2 μm) was prepared using the SPS method. The phase structure of Al_2_CoCrFeNiTi HEAs changes from face-centered cubic to a mixture of face-centered cubic and body-centered cubic as the aluminum molar ratio increases and the core–shell structure becomes more homogeneous. The hardness and fracture toughness also improved, with a Vickers hardness and fracture toughness of 2464.5 MPa and 18.2 MPa·m^1/2^, respectively [123].

The high-temperature mechanical properties of Ti(C,N)-based cermets prepared with Ni-Co and Al_0.3_CoCrFeNi as binder phases are significantly different, and the high-temperature hardness, bending strength, fracture toughness, and modulus of elasticity of the cermets with Ni-Co as the binder phase decreased by about 54%, 45%, 29%, and 36%, respectively, compared with those at room temperature under a temperature of 1000 °C. The high-temperature hardness, bending strength, fracture toughness, and elastic modulus of cermets with the high-entropy alloy as the binder decreased by about 45%, 21%, 28%, and 36%, respectively, compared with the mechanical properties at room temperature. High-entropy alloy binder cermets exhibit good high-temperature mechanical properties, which is mainly due to the low temperature effect on the dislocation slip system [124]. At high temperatures, when stresses are applied to the cermets, dislocations form in the binder and, then, accumulate at the grain boundary edges of the Ti(C,N) exocyclic phase all the way to the Ti(C,N) core, which ultimately produces an overall plastic deformation of the cermets in the stressed region. In addition, the high-entropy alloy binder has a relatively low lamination energy, which can promote the formation of twin crystals. In contrast, the stacking of dislocations has a high layer error energy, which hinders the slip of dislocations [125], thus improving the high-temperature strength of the metal–ceramics.

In the study of Ti(C,N)-WC-Mo_2_C-TaC-AlCoCrFeNi cermet, it was observed that the organization showed both W-rich and W-poor binders. In addition, the surface binder enrichment phenomenon occurs due to the liquid phase migration driving force generated by the decarburization of the cermet surface during the sintering process, which drives the liquid-phase binder to migrate from the interior to the surface. The W-rich and W-poor binder regions with different volume contents in the microstructure of the metal–ceramics generate pressure differences, which exacerbate the enrichment of the binder.

The TiC-HEA cermets prepared by Liu et al. [126] have ultra-high compressive strengths of more than 3000 MPa compared to the compressive strengths of conventional TiC-based cermets of (1790–2210) MPa due to the fine-grain reinforcement and solid-solution strengthening of high-entropy alloys. The two CoCrFeNiCu- Ti(C,N) ceramics have higher fracture toughness and hardness compared to Ni-Ti(C,N) ceramics [115]. The fracture toughness was 8.8 and 9.8 MPa·m^1/2^, respectively, and the hardness was 1726 and 1580 HV, respectively. This was mainly due to the the high-entropy alloying binder phase, which inhibited grain growth and suppressed crack initiation and extension. The Ti(C,N)-based cermets synthesized by Fang et al. [127] using Al_0.3_CoCrFeNi as a binder improved the fracture toughness at room temperature compared to the Ni/Co binder, but the strength decreased. This indicates that the high-entropy binder phase has the ability to toughen the cermet to some extent.

By preparing (Ti,Ta,Nb)(C,N) cermets of FeCoNiCrMn and FeCoNiCrMnAl, it was found that the microhardness gradually increased with increasing temperature for Al-free cermets. However, for the Al-containing cermets, the opposite is true, and the microhardness tends to decrease with increasing sintering temperature. This trend suggests that the presence of Al favors phase segregation at the temperature and the formation of intermetallic compounds. This phase segregation makes the microhardness of the cermets decrease and become brittle [128].

Pötschke et al. [129] passed the HEA binder for new MnFeCoNiCu with different binder values and two different C/N ratios of TiCN hard phases for cermets. In pairs of equimolar MnFeCoNiCu compositions, a small amount of Cu precipitation was found after sintering, and it was necessary to reduce the Cu content by 50% and increase the Ni content by 50% to obtain a single solid solution binder phase. The mechanical properties of TiCN 70/30 cermets with a 16 vol.% Mn_0.2_Fe_0.2_Co_0.2_Ni_0.2_Cu_0.1_ binder phase were found to be comparable to or even better than those of the conventional reference cermets by multiple sets of experiments, with hardness values >1210 HV30 and a fracture toughness of 14.8 MPa·m^1/2^.

### 3.4. Oxidation Resistance and Wear Resistance of High-Entropy Alloy as Binder Phase

The antioxidant property of high-entropy alloys as the binder phase of cermet is mainly due to the elements such as Al and Cr, which can form a dense oxide film on the surface of the cermet and significantly improve the antioxidant property of the cermet. Zhu [130] conducted an in-depth study on the high-temperature antioxidant mechanism of Ti(C,N)-AlCoCrFeNi-based cermets. They found that, in the high-entropy alloy binder phase, the Al and Cr elements would be preferentially oxidized at high temperatures and gradually diffuse and solid-solve into TiO_2_ and WO_3_, and then, AlTiO_5_ and Cr_4_WO_6_ were generated. The formation of these oxides has a low Gibbs free energy and is very stable, which can inhibit oxygen transport and improve the antioxidant performance. Meanwhile, the thermal expansion coefficients of Al_2_O_3_ and Cr_2_O_3_ do not differ much from those of the Ti(C,N) substrate, which reduces the thermal stress tendency of the oxide film and makes the oxide film and the substrate tightly bonded, preventing the contact between the air and the internal substrate and slowing down the oxidation process.

Fang et al. [131] investigated the oxidation behavior of Ti(C,N)-based cermets with Ni-Co and Al_0.3_CoCrFeNi high-entropy alloys as the binder phase at 1000 °C in static air, respectively. They found that Fe_2_O_3_ and Cr_2_O_5_ formed by oxidation i then Al_0.3_CoCrFeNi high-entropy alloy have a strong antioxidant ability, which can slow down the diffusion of oxygen in the cermets, stabilize the oxide layer, and reduce the generation of TiO_2_, greatly improving the high-temperature antioxidant ability.

Li et al. [116] prepared Ti(C,N)-based metal–ceramics by using Ni/Co and FeCoCrNiAl high-entropy alloys as the binder phases, respectively. Ti(C,N)-based cermets were prepared and tested for their mass weight gain versus oxidation time curves in oxidation tests at 1000 °C. The kl values were calculated to be 5.57 × 10^−4^ mg·cm^−2^·s^−1^ for the high-entropy alloy binder and 6.69 × 10^−4^ mg·cm^−2^·s^−1^ for the Ni/Co binder. The kl of the cermets with the high-entropy alloy as the binder had better oxidation resistance than that of the Ni/Co binder cermets under the same conditions.

In addition to oxidation resistance, Ti(C,N)-based cermets with high-entropy alloys as the binder also have good wear resistance at high temperatures. Fang et al. [131] tested the wear resistance of the Al_0.3_CoCrFeNi high-entropy alloy and Ni-Co binder cermets at high temperatures up to 900 °C and found that the incorporation of high-entropy alloys can effectively improve their overall hardness, reduce the hard particle peeling and wear, and lower the friction coefficient, resulting in better wear resistance. Wang et al. [115] prepared different Ti(C,N) cermets (labeled CN, CM, and CG, respectively) using Ni powder, mechanically alloyed CoCrFeNiCu high-entropy alloy powder, and gas-atomized CoCrFeNiCu high-entropy alloy powder, respectively, and found that the width or depth of the abrasion marks on cermets CM and CG were much smaller than those on cermets CM and CG. The width and depth were much smaller than those of metal–ceramics CN, indicating that their high-temperature wear resistance was much higher than that of metal–ceramics CN. Gou et al. [132] added NbC to Ti(C,N)-CoCrFeNi-based metal–ceramics, which can reduce the sintering temperature, increase the densification of the material, and improve its high-temperature wear resistance.

Li et al. [133] tested the wear resistance of Ti(C,N)-FeCoCrNiAl cermets in the temperature range of 200–800 °C and compared them with Ti(C,N)-Ni-Co cermets. The results showed that, when the temperature was lower than 600 °C, there was no significant difference between the wear surfaces of the two cermets, and the wear mechanism was mainly abrasive wear. At temperatures higher than 600 °C, oxidative wear and adhesive wear were the main wear mechanisms. The wear rates of the Ti(C,N)-FeCoCrNiAl cermets were 11.8%, 17%, 39.25%, and 46.7% lower than those of the Ti(C,N)-Ni-Co cermets at 200, 400, 600, and 800 °C, respectively, i.e., the wear rate of the Ti(C,N)-FeCoCrNiAl was lower than that of Ti(C,N)-Ni-Co at different tested temperatures. The wear rate increased and, then, decreased with the increase of the tested temperature. The reason for this is that the FeCoCrNiAl high-entropy alloy has more-excellent resistance to high temperatures compared to the Ni-Co binder.

The Al_0.3_CoCrFeNi-Ti(C,N) cermet prepared by Fang et al. [131] had higher hardness (1137 HV) and fracture toughness (6.46 MPa·m^1/2^) than the Ni-Co binder at 1000 °C. These good mechanical properties at high temperatures are mainly attributed to the addition of high-entropy alloys, which dampen the metal–ceramic sliding system, and the higher oxidation resistance of the high-entropy alloys themselves. Similarly, the FeCoCrNiAl-Ti(C,N)-TiB_2_ cermets prepared by Li et al. [116] had better hardness and oxidation resistance at elevated temperatures than those with Ni-Co binder. Wang et al. [115] investigated the high-temperature wear behavior of Ti(C,N)-CoCrFeNiCu ceramics at 600 and 800 °C. They found that the conventional Ti(C,N)-Ni wear rate was 1.8- to 2.4-times higher than that of the Ti(C,N)-HEA ceramics. The wear mechanism of the Ti(C,N)-Ni ceramics is mainly friction oxide formation and delamination and abrasive wear. In contrast, the wear mechanism of the Ti(C,N)-HEA ceramics is mainly the formation and delamination of the friction oxide layer. This indicates that the Ti(C,N)-HEA ceramics have excellent high-temperature wear resistance. The main reason for this is that the addition of the high-entropy alloy improves the microstructure of the metal–ceramics and increases the hardness and toughness of the matrix. On the other hand, the thermal stability of CoCrFeNiCu at high temperatures stabilizes the binder layer and forms a dense lubricant layer, which improves the high-temperature wear resistance and wear reduction of the ceramics.

The oxidation resistance of metal–ceramics was effectively enhanced by adding ZrO_2_ to the Al_0.3_CoCrFeNi high-entropy alloy. This enhancement is mainly attributed to the high entropy effect and the excellent performance exhibited by the ZrO_2_ whiskers at high temperatures, mainly due to the grain boundary softening. The high grain boundary strength of the metal–ceramics, the hardness and oxidation resistance of the high-entropy binder at elevated temperatures, and the high-temperature synergistic strengthening of the ZrO_2_ whiskers all work together to strengthen the metal–ceramics in a high-temperature environment [134].

The high-entropy alloy binder can significantly improve the high temperature performance of metal–ceramics. This is mainly due to the addition of the high-entropy alloy to change the microstructure of the matrix, refine the grain, and make the phase distribution uniform, thus playing a role in strengthening the matrix. In addition, high-entropy alloys have good oxidation resistance, corrosion resistance, and wear resistance at high temperatures. These characteristics form a dense oxide layer on the substrate, which not only prevents further oxidation of the substrate, but also plays a role in lubrication and wear reduction.

### 3.5. Corrosion of High-Entropy Alloy Binder Phases

High-entropy alloys, as a novel type of multi-subject element alloy, have surpassed the design limitations of conventional alloys based on a single majority of subject elements. This class of alloys has a great potential for improved corrosion resistance, which suggests that they have significant economic and safety benefits for applications in harsh environments. Here, we report on the preparation of FeB-10Mo-12Al0.25FeNiCoCr high-entropy alloys and the enhancement of their corrosion resistance by the addition of Mo elements and corrosion products. At 450 °C, Mo did not undergo eutectic reaction with Zn, but formed corrosion products together with other elements, which consisted mainly of the δ phase and significantly improved the corrosion resistance of the new cermet [135].

## 4. Conclusions and Prospects

- In Ti(C,N)-based cermets, the role of the binder is crucial. In order to further improve the overall performance of metal–ceramics, the study of the binder is particularly important. In recent years, new binder phases, such as intermetallic compounds and high-entropy alloys, have shown some improvement in the mechanical properties, oxidation resistance, corrosion, and wear resistance of Ti(C,N)-based cermet [136]. However, the main direction of the current research is still dominated by experiments and the analysis of the experimental results, and in-depth research on the influencing mechanisms (such as the kinetics, thermodynamics, in situ reflections, etc.) still needs to be strengthened. In addition, the experimental research is time-consuming, and the experimental influencing factors are complex; if we can use computer simulation to assist the experiment, it is expected to greatly save the experimental time and economic costs [20,137].
- Biphasic eutectic CoNiAl alloys can maximize the advantages of each metal as a binder. Therefore, future research directions can include the preparation of such alloys and the in-depth investigation of their properties to further improve the comprehensive use of metal–ceramic properties. Specifically, the preparation process, heat treatment system, microstructure, mechanical properties, physical properties, chemical properties, and corrosion resistance of CoNiAl alloys can be investigated to fully understand the potential of the alloys to be used in metal–ceramics. In addition, aspects such as the interfacial bonding of the alloy with other materials, as well as the stability and oxidation resistance of the alloy at elevated temperatures can also be investigated. These studies are expected to lead to the development of metal–ceramic materials with excellent comprehensive performance to meet the needs of different fields [138].
- Adhesion and wettability between the binder and the ceramic particles is a central issue in the preparation of cermets. However, due to the limited understanding of adhesion, the selection of metal–ceramic materials has always relied on previous literature studies, randomized trials, and rules of thumb. The development of a database detailing experimentally measured wetting angle data under different conditions, coupled with machine learning (ML) algorithms to propose a model for ionic covalent ceramics with respect to metal wettability, would greatly improve design efficiency and reliability [139]. By collecting and organizing a large amount of experimental data, a database on the wetting angle will be established. This database should contain data on the wetting angles of various ionic covalent ceramics and metals under different conditions, which will serve as the basis for the machine learning model. These data will then be used to apply appropriate machine learning algorithms (e.g., linear regression, support vector machines, neural networks, etc.) to train and optimize the model based on specific wettability prediction objectives. This will be a powerful tool for learning from known data and predicting new data and can greatly improve our understanding of wettability for more-effective material design. Finally, with this model, we can predict wettability under different conditions to further optimize the preparation process of metal–ceramic materials and improve their performance and reliability. This will open up new paths for the research and development of metal–ceramic materials, shorten the development cycle, reduce the development cost, and help promote the wider application of metal–ceramic materials in various fields.

## Data Availability

No new data were created nor analyzed in this study. Data sharing is not applicable to this article.
